# Supplementary material for: Diabetic kidney disease in northwest Ethiopia: Prevalence and determinants among adults with type 2 diabetes
Source: PLoS One. 2026 Feb 20;21(2):e0343210. doi: 10.1371/journal.pone.0343210 (PMC12923007; doi:10.1371/journal.pone.0343210)
Supplement: S1 File — (PDF) [file pone.0343210.s001.pdf]

S1 Table. Completed STROBE checklist for cross-sectional studies – DKD prevalence in T2DM patients

This checklist was elaborated using formal items recommended for cross-sectional studies from STROBE statement (https://www.strobe-statement.org).

	Item No	Recommendation	Page No	Comments and quotes
**Title and abstract**	1	(*a*) Indicate the study's design with a commonly used term in the title or the abstract	1 and 2	Study design is indicated in the Methods section of the abstract' Cross-sectional institution-based study'
		(*b*) Provide in the abstract an informative and balanced summary of what was done and what was found	2	Described in the results and conclusion section of the abstract
**Introduction**				
Background/rationale	2	Explain the scientific background and rationale for the investigation being reported	4,5,6	Existing literature and rationale are stated in the introduction section
Objectives	3	State specific objectives, including any prespecified hypotheses	6	A statement at the end of the introduction specifies the objectives. "The aim of this study was to comprehensively assess the prevalence of DKD in patients with T2DM by using different diagnostic markers..."
**Methods**				
Study design	4	Present key elements of study design early in the paper	6	The study design is first section of the methods- 'Design and setting' section on methods
Setting	5	Describe the setting, locations, and relevant dates, including periods of recruitment, exposure, follow-up, and data collection	6 and 7	All stated under ' study design and setting' section
Participants	6	(*a*) Give the eligibility criteria, and the sources and methods of selection of participants	7	Inclusion/exclusion criteria described under ' Source and study population' and 'sampling method' subsection on methods
Variables	7	Clearly define all outcomes, exposures, predictors, potential confounders, and effect modifiers. Give diagnostic criteria, if applicable	8 and 9	This is described under' variables and operational definitions' section
Data sources/ measurement	8*	For each variable of interest, give sources of data and details of methods of assessment (measurement). Describe comparability of assessment methods if there is more than one group	9 and 10	Data collected via questionnaire from interview and chart review. Details of measurement of cr, cystatin C albuminuria, A1c, and eGFR calculation and

				diagnosis of DKD described under' data collection methods and procedures' section
Bias	9	Describe any efforts to address potential sources of bias	7,9,10	To avoid selection bias, we set clear inclusion/exclusion criteria, Selected through systemic random sampling method. To avoid measurement bias, we use similar lab and machines to measure cr, cystatin C and albuminuria. Multivariate analysis done to avoid confounding bias
Study size	10	Explain how the study size was arrived at	7	It was calculated based on findings from previous studies and certain assumptions- It is described under' sample size and sampling technique' section
Quantitative variables	11	Explain how quantitative variables were handled in the analyses. If applicable, describe which groupings were chosen and why	8,9	Definitions of all categories for variables are presented in the 'variables and operational definition 'section
Statistical methods	12	(*a*) Describe all statistical methods, including those used to control for confounding	11	Described under the 'statistical analysis ' section of the method part
		(*b*) Describe any methods used to examine subgroups and interactions	N/A	Not Applicable
		(*c*) Explain how missing data were addressed	11	No missing values were observed in the dataset, and all cases were included in the analysis
		(*d*) If applicable, describe analytical methods taking account of sampling strategy	N/A	Not Applicable
		(*e*) Describe any sensitivity analyses	N/A	Not Applicable
**Results**				
Participants	13*	(a) Report numbers of individuals at each stage of study—eg numbers potentially eligible, examined for eligibility, confirmed eligible, included in the study, completing follow-up, and analysed	13	This is described at the beginning of result section<br>A total of 210 T2DM who met our study criteria were approached, and 204 responded positively (response rate = 97.1%).
		(b) Give reasons for non-participation at each stage	?	We did not collect specific reasons for the small number of non-respondents. However, the low non-response rate makes it unlikely to significantly impact the findings.

		(c) Consider use of a flow diagram	N/A	It was deemed unnecessary to use flow diagram
Descriptive data	14*	(a) Give characteristics of study participants (eg demographic, clinical, social) and information on exposures and potential confounders	13	Tables 1, Table, and Figure 1 describes characteristics of study participants
		(b) Indicate number of participants with missing data for each variable of interest	13,14	The total number of recorded data points for the participants is indicated in the header of each table. Additionally, the frequency and proportion of each variable are presented in the tables
Outcome data	15*	Report numbers of outcome events or summary measures	13,14	All the number of outcome events and relevant summary measures are reported in the results section in tables
Main results	16	(*a*) Give unadjusted estimates and, if applicable, confounder-adjusted estimates and their precision (eg, 95% confidence interval). Make clear which confounders were adjusted for and why they were included	14	Unadjusted and adjusted estimates are described in Table 4
		(*b*) Report category boundaries when continuous variables were categorized	13, 14	Category boundaries are displayed in the variable headings of the tables, where applicable (e.g., age groups, sex, duration of DM, comorbidity type)
		(*c*) If relevant, consider translating estimates of relative risk into absolute risk for a meaningful time period	N/A	N/A
Other analyses	17	Report other analyses done—eg analyses of subgroups and interactions, and sensitivity analyses	N/A	N/A
**Discussion**				
Key results	18	Summarise key results with reference to study objectives	15	Key findings are outlined at the start of the discussion section
Limitations	19	Discuss limitations of the study, taking into account sources of potential bias or imprecision. Discuss both direction and magnitude of any potential bias	17	The discussion of the limitations of this study is provided in the 'Limitations of the study' section
Interpretation	20	Give a cautious overall interpretation of results considering objectives, limitations, multiplicity of analyses, results from similar studies, and other relevant evidence	18	The interpretation is presented in the 'Conclusion' section, taking into account all relevant references from prior studies and the study's limitations
Generalisability	21	Discuss the generalisability (external validity) of the study results	18	This study used good quality primary data collected using validated markers for the dx of DKD. However,

				as this is an institution-based study, we recognize the limitation related to generalizability and we mentioned this in 'Limitation' section
**Other information**				
Funding	22	Give the source of funding and the role of the funders for the present study and, if applicable, for the original study on which the present article is based	19	Funding information is provided in the paper

*Give information separately for exposed and unexposed groups.

Note: An Explanation and Elaboration article discusses each checklist item and gives methodological background and published examples of transparent reporting. The STROBE checklist is best used in conjunction with this article (freely available on the Web sites of PLoS Medicine at http://www.plosmedicine.org/, Annals of Internal Medicine at http://www.annals.org/, and Epidemiology at http://www.epidem.com/). Information on the STROBE Initiative is available at www.strobe-statement.org.
